# Multinational Study to Assess Stress Levels Among the Health Care Workers of Radiation Oncology Community at the Outset of the COVID-19 Pandemic

**DOI:** 10.1200/GO.20.00647

**Published:** 2021-04-06

**Authors:** Tabassum Wadasadawala, Anuj Kumar, Sarbani Ghosh Laskar, Soehartati Gondhowiardjo, Smruti Mokal, Savita Goswami, Angela Giselvania, Rakesh Kapoor, Abhijit Das, Satyajit Pradhan, Lincoln Pujari, Bibek Acharya, Sandhya Chapagain, Umesh Mahantshetty, Rohit Vadgaonkar, Qazi Mushtaq Hussain, Kamal Akbarov, Jai Prakash Agarwal

**Affiliations:** ^1^ Department of Radiation Oncology, Tata Memorial Centre, Homi Bhabha National Institute, Mumbai, India; ^2^ Department of Radiation Oncology, Dr. Cipto Mangunkusumo National General Hospital, Jakarta, Indonesia; ^3^ Department of Biostatistics, Tata Memorial Centre, Homi Bhabha National Institute, Mumbai, India; ^4^ Department of Clinical Psychology, Tata Memorial Centre, Homi Bhabha National Institute, Mumbai, India; ^5^ Department of Radiation Oncology, Homi Bhabha Cancer Hospital, Sangrur, India; ^6^ Department of Radiation Oncology, Homi Bhabha Cancer Hospital, Varanasi, India; ^7^ Department of Radiation Oncology, National Academy of Medical Sciences, Bir Hospital, Kathmandu, Nepal; ^8^ Department of Radiation Oncology, Homi Bhabha Cancer Hospital and Research Centre, Visakhapatnam, India; ^9^ Department of Radiation Oncology, National Institute of Cancer Research and Hospital, Dhaka, Bangladesh; ^10^ Department of Nuclear Sciences and Applications, International Atomic Energy Agency, Vienna, Austria

## Abstract

**PURPOSE:**

To evaluate stress levels among the health care workers (HCWs) of the radiation oncology community in Asian countries.

**METHODS:**

HCWs of the radiation oncology departments from 29 tertiary cancer care centers of Bangladesh, India, Indonesia and Nepal were studied from May 2020 to July 2020. A total of 758 eligible HCWs were identified. The 7-Item Generalized Anxiety Disorder, 9-Item Patient Health Questionnaire, and 22-Item Impact of Events Scale-Revised were used for assessing anxiety, depression, and post-traumatic stress disorder. Univariate and multivariate analysis was done to identify the causative factors affecting mental health.

**RESULTS:**

A total of 758 participants from 794 HCWs were analyzed. The median age was 31 years (IQR, 27-28). The incidence of moderate to severe levels of anxiety, depression, and stress was 34.8%, 31.2%, and 18.2%, respectively. Severe personal concerns were noticed by 60.9% of the staff. On multivariate analysis, the presence of commonly reported symptoms of COVID-19 during the previous 2 weeks, contact history (harzard ratio [HR], 2.04; CI, 1.15 to 3.63), and compliance with precautionary measures (HR, 1.69; CI, 1.19 to 2.45) for COVID-19 significantly predicted for increasing anxiety (HR, 2.67; CI, 1.93 to 3.70), depression (HR, 3.38; CI 2.36 to 4.84), and stress (HR, 2.89; CI, 1.88 to 4.43) (*P* < .001). A significant regional variation was also noticed for anxiety, stress, and personal concerns.

**CONCLUSION:**

This survey conducted during the COVID-19 pandemic revealed that a significant proportion of HCWs in the radiation oncology community experiences moderate to severe levels of anxiety, depression, and stress. This trend is alarming and it is important to identify and intervene at the right time to improve the mental health of HCWs to avoid any long-term impacts.

## INTRODUCTION

### 

The pervading COVID-19 of epic proportions, initially announced as a Public Health Emergency of International Concern, was later declared as a pandemic on March 11, 2020, by the WHO.^1^ The phenomenal increase in the number of COVID cases has put tremendous pressure on government-run health care systems in almost every country across the world. India, presently the second worst-hit nation in the world, with more than 6 million positive cases, presents a steadily rising diagrammatic trend.^2^ The state of Maharashtra in India is the most affected, with Mumbai recording the highest number of victims. The Mumbai metropolitan city records around 1,100 to 1,900 COVID cases per day, with a steep increase in the number of affected people and related deaths across the country.^3^ Among other countries in South East Asia, Indonesia has reported more than 270,000 affected cases and more than 10,000 deaths as of now. Similarly, among neighboring countries, Nepal has recorded 70,000 cases with more than 400 deaths. Bangladesh of late reported about 350,000 cases and more than 5,000 deaths.^4^

**CONTEXT**

**Key Objective**
To evaluate the mental health outcomes of health care workers (HCWs) of the radiation oncology community in Asian countries during the COVID-19 Pandemic.
**Knowledge Generated**
In this cross-sectional country stratified survey of 758 HCWs, the incidence of moderate to severe levels of anxiety, depression, and stress was 34.8%, 31.2%, and 18.2%, respectively. Development of COVID-related symptoms, exposure to COVID cases, and noncompliance to safety precautions significantly increased the levels of anxiety, depression, and stress.
**Relevance**
A striking regional variation was noticed, and the severity of psychologic parameters was higher in HCWs of Bangladesh and Indian participants compared with Indonesian and Nepalese. It is important to understand the mental health of HCWs who play a crucial role in fighting this pandemic and ensure that appropriate and timely interventions are undertaken to mitigate the lingering mental sequelae of HCWs.

The Tata Memorial Centre (TMC) is a premier grant-in-aid health care institution under the Department of Atomic Energy, Government of India. Located at the epicenter of Mumbai metropolis, it caters to patients with cancer across the globe, using state-of-the-art treatment procedures and physicking to more than 70,000 cases every year. The Radiation Oncology department at TMC, Mumbai, and its sister institutions in various parts of India cater to cancer care services across the country.^5^ The department comprises consultant radiation oncologists, medical physicists, radiation therapy technologists, nurses, trainees, and social workers. The radiation oncology community of neighboring countries of India including Bangladesh, Nepal, and Indonesia has analogous facilities, workflow, and departmental staff.

The COVID-19 pandemic has affected cancer care services provided by all hospitals especially because of the restrictions imposed by the nation-wide lockdown aimed at impeding the spread of the contagion. Under such circumstances, the timely implementation of various administrative policies to enable the continuation of cancer care along with preparations for effectively handling this medical emergency is of paramount importance. Notably, both patient-directed and employee-directed measures that minimize the risk of contracting COVID-19 should be adopted. However, the escalating trend in the number of positive cases, social stigma, and fear of family members contracting the disease adds to the psychologic and social trauma, which has a demoralizing effect on the mental health of the cancer care providers. These are important problems that have to be addressed and comprehended for effective medical management of the pandemic.

The 7-item Generalized Anxiety Disorder (GAD-7) scale and 9-item Patient Health Questionnaire (PHQ-9) are routinely used in clinics to assess anxiety and depression, whereas the 22-item Impact of Events Scale-Revised (IES-R) can analyze the psychologic trauma and assess the post-traumatic stress disorder. All these instruments have been validated and authoritatively used for assessing psychologic disorders.^6-9^ These questionnaires were also used to evaluate the mental well-being of health care workers (HCWs) who continued to serve patients during the ongoing pandemic.^10^ This study is directed at analyzing stress levels and burnout among the radiation oncology community in tertiary cancer care facilities in four Asian countries of Bangladesh, India, Indonesia, and Nepal.

## METHODS

Approval from the Institutional Ethics Committee and registration on the Clinical Trial Registry of India was undertaken before the initiation of the study. This is a cross-sectional, survey-based, country-stratified study. Cancer care centers with facility of a radiation oncology department were considered eligible for participation. HCWs in all TMC hospitals of India and various centers in Bangladesh, Indonesia, and Nepal were approached. Informed consent was obtained from all participants in the 29 centers (Appendix). The GAD-7, PHQ-9, and IES-R questionnaires were provided with the help of Google forms, and responses were recorded for analysis. Hand-written forms were taken from participants who were unable to fill the Google forms. No direct contact information was sought from the study participants to keep the survey anonymized.

### Participants

The staff of all cadres (oncologists, physicists, radiation therapy technologists, nurses, administrative staff, and allied workers) participated in the study. Participants were enrolled from May 16, 2020, till July 25, 2020. All HCWs in the department of various centers were requested to fill in the questionnaires with assured confidentiality of the information provided. Out of the 399 forms served in five TMC hospitals across India, 397 consented for the study. Twenty-two centers from Indonesia along with one center each from Bangladesh and Nepal contributed to 395 HCWs, making a total of 794 HCWs (Data Supplement).

### Outcomes and Measures

Severity of anxiety, depression, and post-traumatic stress disorder were assessed from the GAD-7, PHQ-9, and IES-R scores. For GAD-7, scores 0-4 represent minimal, 5-9 mild, 10-14 moderate, and 15-21 severe anxiety. For PHQ-9, scores 0-4 represent none, 5-9 mild, 10-14 moderate, 15-19 moderately severe, and 20-27 severe depression. For IES-R scored from 0 to 88, a total score of 24 or more post-traumatic stress disorder is a clinical concern.^11,12^

### Statistical Analysis

Data analysis was done using Statistical Package for Social Sciences software, Version 25. Univariate analysis was done to identify the important causative factors for anxiety, depression, and stress, and significant factors were then entered into a multivariate Cox proportional hazards model and expressed as hazard ratio (HR) with a 95% CI. Any *P* value ≤ .05 was considered statistically significant.

## RESULTS

### Demographic Profile

Out of the 794 total consented participants, 36 participants with incomplete responses were excluded and hence 758 HCWs were considered for the final analysis. A basic sociodemographic profile was collected from all participants. This included factors such as age, sex, marital status, number of household members, rooms in the household, distance from home to workplace, educational level, occupation, comorbidities, history of smoking or any COVID-19–related symptoms, contact history in the prior 14 days, lifestyle changes, and personal concerns. The sociodemographic details of all the 758 participants are shown in Table 1.

**TABLE 1:**
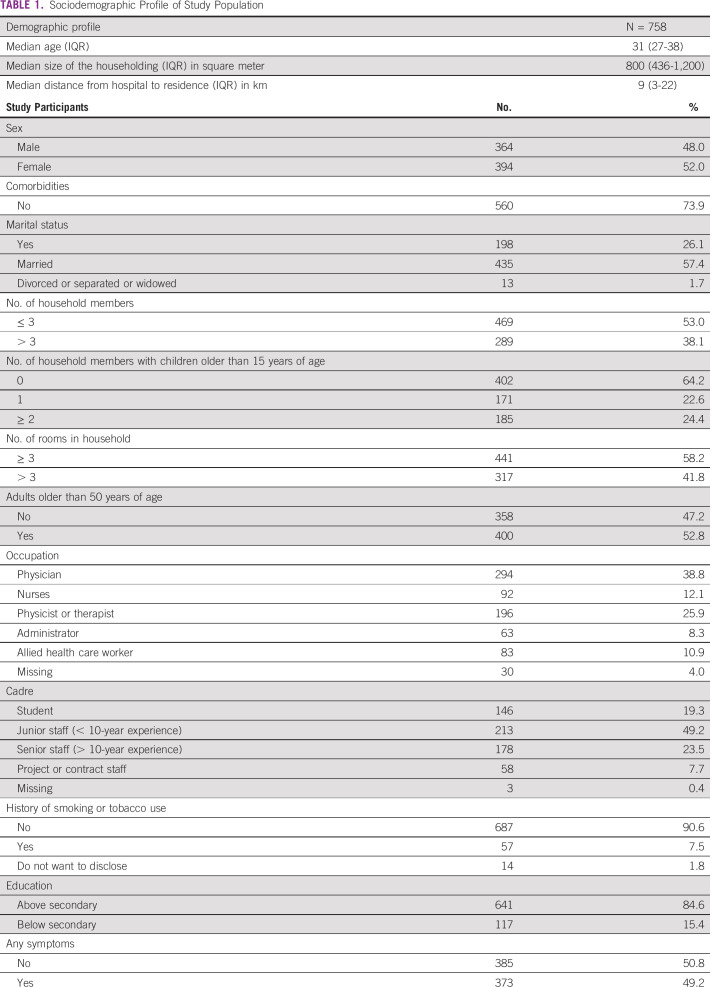
Sociodemographic Profile of Study Population

### COVID-19 Symptoms

**FIG 1:**
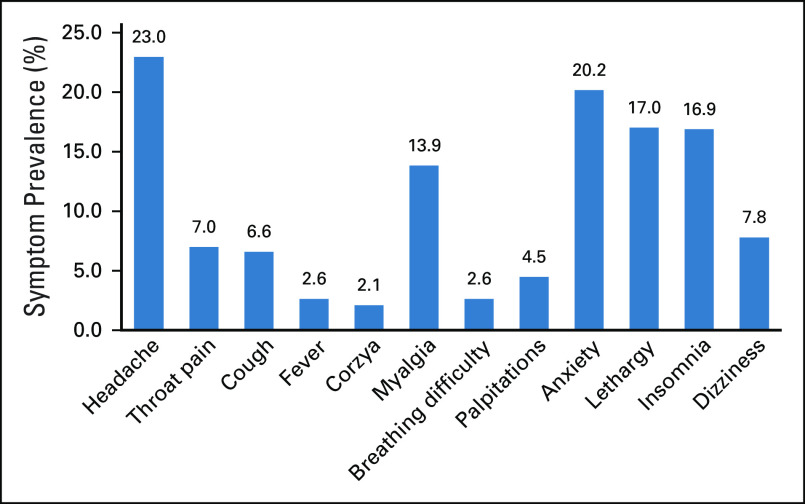
Percentage of symptom prevalence.

COVID-19 is known to cause a wide variety of symptoms ranging from mild symptoms to severe illness. Symptoms mostly appear 2 to 14 days after exposure to the virus. For our study, the recall period for COVID-19–related symptoms was up to 2 weeks before the date of filling of the forms. The percentage of symptom prevalence was different in different countries. In Bangladesh, anxiety was the most commonly reported symptom seen in all the participants (100%). In India, headache and anxiety were most commonly reported (21.5%), whereas in Indonesia and Nepal, myalgia (22.9%) and coryza (100%) were more frequent. The percentage of symptom prevalence is shown in Figure 1.

### COVID-19 Contact History and Precautionary Measures

**FIG 2:**
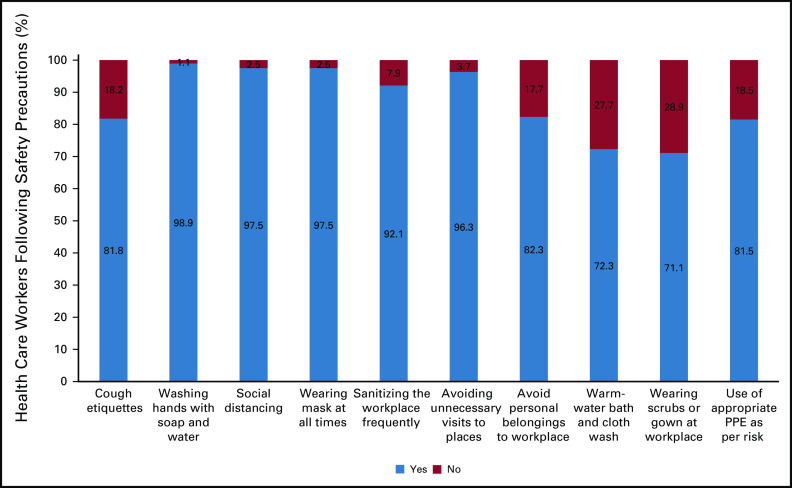
Percentage of health care workers following safety precautions.

History of contact is one of the most important factors that can increase the level of anxiety and stress. Across different countries, participants from Indonesia had maximum contact at the workplace (47.3%). Direct and indirect contact with a COVID-positive case was higher in HCWs from Bangladesh (29.4% and 64.7%, respectively). Recent history of testing was higher in participants from Indonesia (25.4%), whereas HCWs from Bangladesh had higher rates of quarantine (17.6%) (Data Supplement). It is very important for health care workers to follow all the possible safety precautions during this pandemic. Cough etiquettes, frequent handwashing with soap and water, social distancing, wearing a mask, sanitizing the workplace, avoiding unnecessary visits, shunning personal belongings at workplace, mandatory warm-water bath and cloth wash, wearing scrubs or gown at workplace, and use of appropriate Personal Protective Equipment (PPE) as per risk are some of the precautions frequently talked about and explained to the HCWs. Among all the safety precautions, the measure not adhered to by a significant portion of the participants (28.9%) was not using gowns or scrubs at the workplace. The use of scrubs during work hours can prevent exposure and transmission through fomites during the transit back from work and at home. The safety measures and the percentage of HCWs practicing them are shown in Figure 2.

### Anxiety, Depression, and Stress Scores

**FIG 3:**
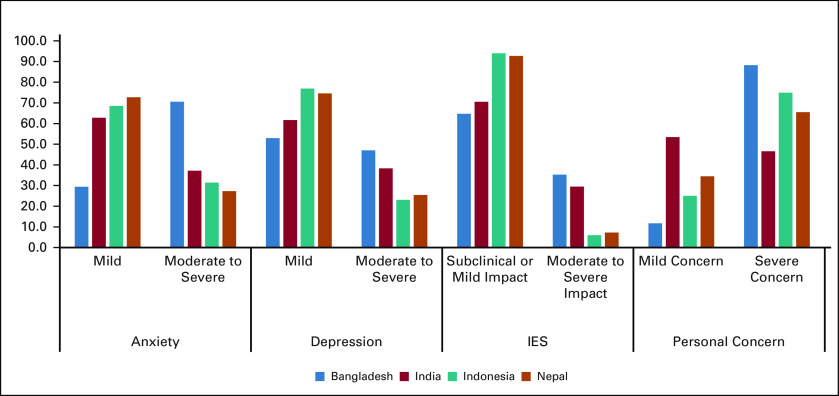
Country-wise percentage incidence of mental health outcomes. IES, Impact of Events Scale.

For evaluation of mental health outcomes of this pandemic in all the participant countries, levels of anxiety, depression, stress, and personal concerns were scrutinized. Among the countries, HCWs from India, Indonesia, and Nepal had predominantly mild symptoms of anxiety and depression, whereas the majority of the participants from Bangladesh had moderate to severe levels of anxiety and stress. Subclinical or mild impact based on IES scores was seen in a majority of the participants. It was seen that personal concerns were of more severe proportion in Bangladesh followed by Indonesia, Nepal, and India. Likelihood of contracting COVID-19 during the current outbreak, anxiety about family members getting infected, worry about a young child getting the contagion, fear of being isolated, feelings of uncertainty and social stigmatization, and fear of family care in case of self-isolation were the questions that were asked to all the participants. Figure 3 shows the country-wise distribution of the percentage incidence of anxiety, depression, post-traumatic stress disorder, and personal concerns.

### Univariate and Multivariate Analysis

In univariate analysis, the presence of comorbidities, more than three household members, smoking, history of symptoms, and participants from Bangladesh had moderate to severe levels of anxiety. For depression, median age, unmarried individuals, medical staff, student, history of symptoms, contact with a confirmed or suspect case, not following precautions correctly, and HCWs from Bangladesh had moderate to severe issues. Age, unmarried individuals, history of symptoms, contact with a confirmed or suspect case, and Bangladeshi participants had moderate to severe impact on stress levels. Moderate to severe personal concerns correlated significantly with age, married individuals, more than three household members, allied workers, senior staff, presence of comorbidities, smoking, history of symptoms, and HCWs from Bangladesh (univariate analysis, Data Supplement).

For multivariate analysis, smoking (HR, 1.83 [1.02-3.25]; *P* = .041), history of symptoms (HR, 2.67 [1.93-3.70]; *P* < .0001), Bangladesh (HR, 3.74 [1.08-12.94]; *P* = .037), and India (HR, 2.00 [1.03-3.86]; *P* = .040) correlated for higher levels of anxiety. For depression, history of symptoms (HR, 3.38 [2.36-4.84]; *P* = .000] and not following precautions (HR, 1.69 [1.16-2.45]; *P* = .006) had significant correlation. For stress, history of symptoms (HR, 2.89 [1.88-4.43]; *P* = .000), history of contact (HR, 2.04 [1.15-3.63]; *P* = .016), and HCWs from India (HR, 5.87 [2.03-17.00]; *P* = .001) had significant impact. Age (HR, 0.97 [0.94-1.00]; *P* = .043), married individuals (HR, 1.88 [1.18-2.98]; *P* = .008), more than three household members (HR, 1.90 [1.32-2.72]; *P* = .001), < 15 years (HR, 1.71 [1.15-2.53]; *P* = .008), student (HR, 0.28 [0.12-0.62]; *P* = .002), junior staff (HR, 0.46 [0.24-0.88]; *P* = .019), symptoms (HR, 1.86 [1.31-2.64]; *P* = .001), and HCWs from Indonesia (HR, 2.78 [1.37-5.66]; *P* = .005) had severe concerns (Multivariate analysis, Table 2).

**TABLE 2:**
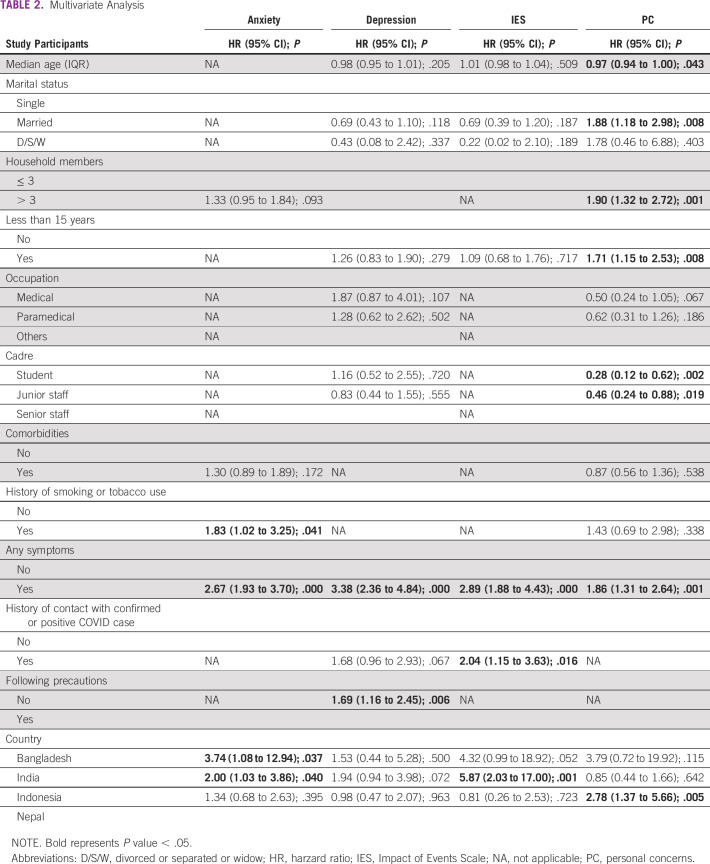
Multivariate Analysis

## DISCUSSION

The COVID-19 pandemic has drastically metamorphosed the day-to-day life of every individual. All of a sudden, the medical profession, its work environment, and above all the psychologic perceptions of patient and self-care issues have undergone an exceptional transformation. The unanticipated and rapid spread of this contagion has problematized and stressed the health care systems across the world.^13^ Increased risk of exposure to the virus during work and daily transit to workplaces potentially exacerbates the problem of burnout and psychologic distress among HCWs and demands compliance with the precautionary measures.^14^ There are myriad causes for anxiety among health care providers, which include the inadequacy of personal protective equipment, changes in the working hours and shifts, access to testing, risk of infecting family members and colleagues at the workplace, and child care compulsions because of shutdown of the educational institutions along with an increase in demands from the domestic front to meet family and social commitments.^15,16^

HCWs are found to be susceptible to fear, anxiety, depression, insomnia, and other mental health problems during the time of a pandemic.^17,18^ In a meta-analysis of 13 studies with more than 33,000 health care workers, the prevalence of anxiety was 23.2% and that of depression was 22.8%.^19^ It is of utmost importance to address the concerns of the frontline medical workers and it is also observed that the risk of reporting COVID-19 positivity is increased among them.^20^ Confidence-building exercises, appropriate measures to allay concerns, and reassurance during this pandemic with regular screening for stress, anxiety, and depression should be a priority. The exposure of HCW to a COVID-positive patient is likely to aggravate stress and associated mental health problems.^21^ The psychologic impact of isolation and quarantine also requires careful redressal.^22^

The current study, to our knowledge, is the largest multinational study in Asian countries performed in HCWs from leading national institutes in the field of oncology who are significantly affected by the COVID-19 pandemic. As the immune-suppressive state in patients with cancer poses them at an increased risk of acquiring the contagion, it also results in greater exposure of the HCW involved in cancer care. Moreover, the relative clustering of the tertiary cancer centers in the metropolitan cities that are worst hit by COVID-19 further leads to greater risk both for the patient as well as the staff. In this way, the results from the current study are generalizable to most of the radiation departments and radiation workers involved in cancer care. As radiotherapy involves daily fractionated treatment over few weeks, the radiation workers are exposed to the patients as well as care givers for a prolonged duration of time, which exacerbates the risk of infection from both symptomatic and asymptomatic carriers.

Of particular interest in our study was that the levels of anxiety, depression, and stress were assessed for all the study participants. It was seen that 34.8% of the population had moderate to severe anxiety, 31.2% had moderate to severe depression, and 18.2% with moderate to severe post-traumatic stress disorder. This seems to be higher than that reported earlier in the meta-analysis, which included 12 studies from China and one from Singapore. This could be because of the difference in the instruments used and the setting as our study included all cadres of the radiation oncology community, whereas the meta-analysis included only doctors and nurses of any discipline.^19^ A review of various conducted studies among health care workers is shown in the Data Supplement.

It was noticed that a large majority (60.9%) of the participants had severe personal concerns. The incidence of personal concern was almost twice in married workers who had children and larger families. This correlation seems justified considering the anxiety of transmitting the infection unknowingly to the family members especially in the extremes of age. At the same time, the concern of home-care in the event of self-isolation for work-place exposure to high-risk cases was the dominant component of personal concern.

Although multiple risk factors as shown in the Data Supplement were found to increase anxiety, depression, and stress on univariate analysis, the development of COVID-related symptoms, history of contact, and not following precautionary measures were correlated on multivariate analysis. The severity of psychologic parameters was higher in Bangladeshi and Indian participants compared with Indonesian and Nepalese. The possible reasons could be that majority of the Indian participants were from TMC, Mumbai, which is a large tertiary cancer center where the COVID-19 pandemic is still at its peak compared with the other peripheral centers.

The pandemic is expected to usher in a second surge in the incidence of mental illness and this will undermine the psychosocial protocols put in place for impeding the multifarious disasters of the COVID-19 pandemic. High priority is currently being bestowed on the physical and emergency management of the COVID-19 pandemic; however, as noticed from the study results, the pandemic is going to leave a huge impact on the psychologic health of the people, especially those involved in the delivery of cancer care. Hence, it becomes the cardinal responsibility of the organization to cater to the unsaid needs of providing psychologic and concomitant support to the HCWs. However, the HCWs should be enlightened to readily avail such services irrespective of the social stigma associated with it.

To address psychosocial distress, feasible measures broadly delineated should be considered appropriately as per the intensity or level of distress. Mild distress should be considered as a symptomatic alarm. Preventive measures should be taken such as enhancing emotional support; empathetic sharing within the group; psychotherapeutic interventions in a close group setting. Moderate to severe level of distress will require individual attention and referral to mental health professionals for systematic management of symptoms and close monitoring. The practice of self-care, relaxation, meditation, hobbies, and engagements should be reinforced for stress and anxiety management. Maintaining a compassionate atmosphere by providing need base support appropriately by colleagues, authorities, or institutions within the group will help to resolve psychosocial stressors.

In this study, the cross-sectional survey for assessing the burden of mental health problems was undertaken. Structured intervention strategies were not specifically designed or integrally included in the protocol. However, participants were encouraged to avail the institutional services for the psychologic interventions as per need. The study results have undermined the need of including counseling and psychologic services in the pandemic management strategies to safeguard the mental health of the health professionals. Second, repeat assessments and interventions at regular intervals will help in the redressal of the mental health problems. This is planned as the second assessment in future as the secondary end point of the study.

In conclusion, in this large study across various cadres of the radiation oncology personnel who have constant exposure, moderate to severe levels of anxiety and depression was noticed in one third of the workforce, whereas moderate to severe stress was documented in one fifth. The development of COVID-related symptoms, history of contact, and noncompliance to precautionary measures were most significantly correlated with increasing levels of anxiety, depression, and stress. The study concludes that appropriate and timely interventions in the backdrop of serious concerns are to be carried out to mitigate the lingering mental sequelae of the pandemic to health care workers.

## Appendix

### List of Participating Centers

#### Bangladesh (n = 1).

National Institute of Cancer Research and Hospital, Dhaka

#### India (n = 5).

1. Tata Memorial Hospital, Mumbai
2. Advanced Centre for Treatment Research and Education in Cancer, Kharghar, Navi Mumbai
3. Homi Bhabha Cancer Hospital, Varanasi
4. Homi Bhabha Cancer Hospital, Visakhapatnam
5. Homi Bhabha Cancer Hospital, Sangrur

#### Indonesia (n = 22).

1. Cancer Center, Adi Husada Hospital, Surabaya
2. Adam Malik Hospital, Medan
3. Awal Bros Cancer Center, Pekanbaru
4. RSUD Al Ihsan, Bandung
5. Kandou Hospital, Manado
6. Murni Teguh Radiotherapy Center
7. Mayapada Hospital
8. Santosa Hospital, Bandung Kopo, Bandung
9. Dr. Ramelan Navy Hospital, Surabaya
10. Dr. Moewardi General Hospital, Surakarta
11. Arifin Achmad General Hospital, Riau
12. Dr. Soetomo General Academic Hospital
13. Gatot Soebroto Central Army Hospital
14. Ken Saras Hospital, Semarang
15. Mohammad Hoesin General Hospital, Palembang
16. Persahabatan Hospital, Jakarta
17. Indriati Hospital, Sukoharjo
18. Sardjito General Hospital, Yogyakarta
19. Dr. H. Abdul Moeloek General Hospital
20. Dharmais Cancer Center Hospital, Jakarta
21. MRCCC Siloam Semanggi, Jakarta
22. Dr. Cipto Mangunkusumo National General Hospital, Jakarta

#### Nepal.

National Academy of Medical Sciences, Bir Hospital, Kathmandu
